# Supplementary material for: Mineralization Profile of Annexin A6-Harbouring Proteoliposomes: Shedding Light on the Role of Annexin A6 on Matrix Vesicle-Mediated Mineralization
Source: Int J Mol Sci. 2022 Aug 11;23(16):8945. doi: 10.3390/ijms23168945 (PMC9409191; doi:10.3390/ijms23168945)
Supplement: Supplementary file 1 [file ijms-23-08945-s001.zip › ijms-1792755-supplementary.pdf]

Supplementary Figures

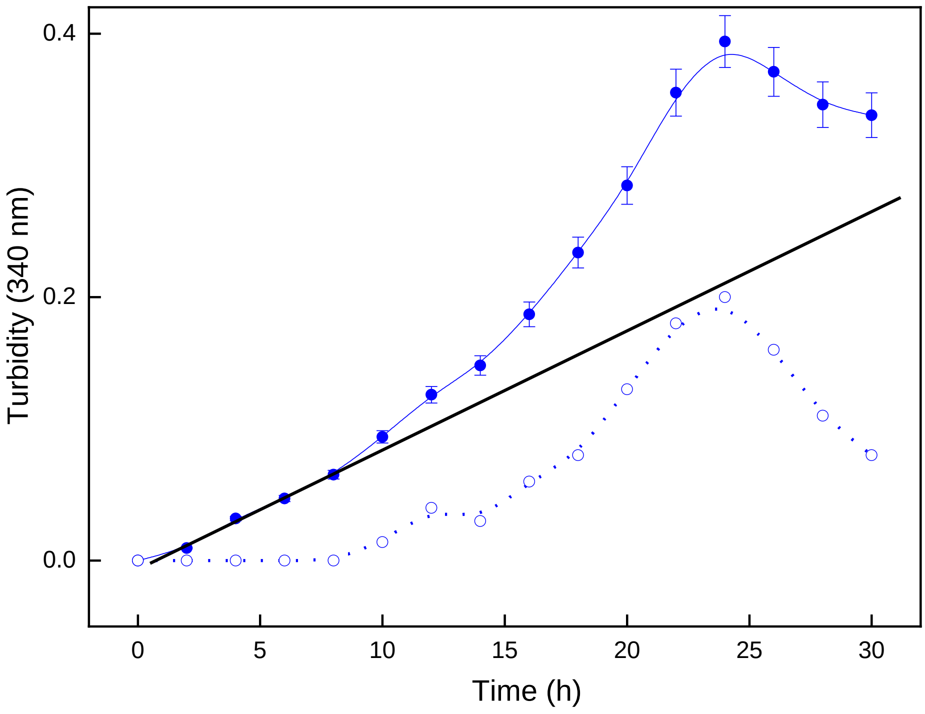

Figure S1: Mineralization curves of proteoliposomes constituted by DPPC harboring AnxA6 with PS-CPLX nucleator in the absence of collagen type-I. Continuous line and solid point is the change with time of the value of absorbance at 340 nm (Figure 2A), dotted line and open symbols are the changes in the absorbance adjusted by the subtraction of the data of black line as considered as monotonic mineralization. For details see results and discussion section.

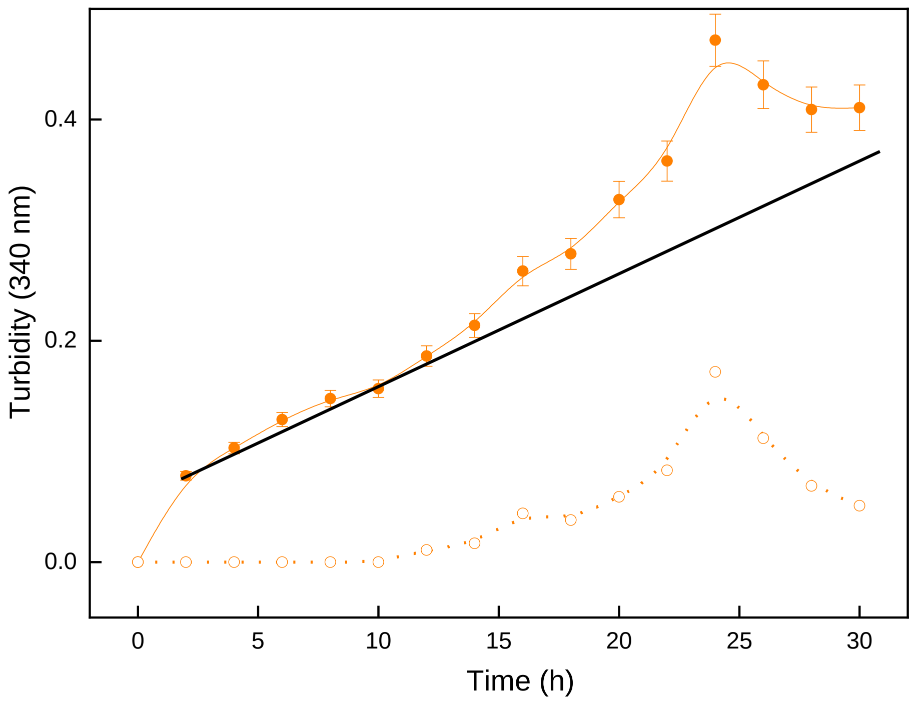

Figure S2: Mineralization curves of proteoliposomes constituted by 9:1 DPPC:DPPS harboring AnxA6 with PS-CPLX nucleator in the absence of collagen type-I. Continuous line and solid point is the change with time of the value of absorbance at 340 nm (Figure 2A), dotted line and open symbols are the changes in the absorbance adjusted by the subtraction of the data of black line as considered as monotonic mineralization. For details see results and discussion section.

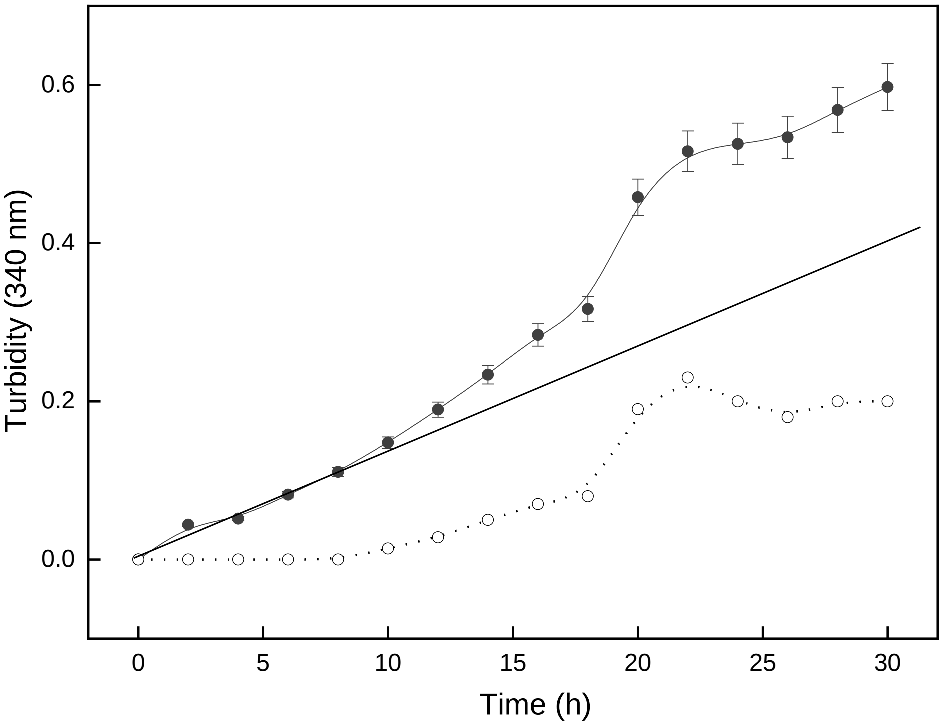

Figure S3: Mineralization curves of proteoliposomes constituted by 5:4:1 DPPC:Chol:DPPS harboring AnxA6 with PS-CPLX nucleator in the absence of collagen type-I. Continuous line and solid point is the change with time of the value of absorbance at 340 nm (Figure 2A), dotted line and open symbols are the changes in the absorbance adjusted by the subtraction of the data of black line as considered as monotonic mineralization. For details see results and discussion section.
